# Internest food sharing within wood ant colonies: resource redistribution behavior in a complex system

**DOI:** 10.1093/beheco/arv205

**Published:** 2015-11-30

**Authors:** Samuel Ellis, Elva J.H. Robinson

**Affiliations:** ^a^Department of Biology, University of York, York YO10 5DD, UK and; ^b^York System for Complex Systems Analysis, University of York, York YO10 5GE, UK

**Keywords:** behavioral plasticity, foraging, *Formica lugubris*, polydomy, social organization, wood ants.

## Abstract

Resource sharing is an important cooperative behavior in many animals. Sharing resources is particularly important in social insect societies, as division of labor often results in most individuals including, importantly, the reproductives, relying on other members of the colony to provide resources. Sharing resources between individuals is therefore fundamental to the success of social insects. Resource sharing is complicated if a colony inhabits several spatially separated nests, a nesting strategy common in many ant species. Resources must be shared not only between individuals in a single nest but also between nests. We investigated the behaviors facilitating resource redistribution between nests in a dispersed-nesting population of wood ant *Formica lugubris*. We marked ants, in the field, as they transported resources along the trails between nests of a colony, to investigate how the behavior of individual workers relates to colony-level resource exchange. We found that workers from a particular nest “forage” to other nests in the colony, treating them as food sources. Workers treating other nests as food sources means that simple, pre-existing foraging behaviors are used to move resources through a distributed system. It may be that this simple behavioral mechanism facilitates the evolution of this complex life-history strategy.

## Lay Summary

Wood ant workers forage to other nests in their spatially dispersed colonies. Ant colonies can be dispersed between several different nests. Sharing resources between these nests is an important challenge for these dispersed colonies. We show that in dispersed colonies of the wood ant *Formica lugubris*, resources are redistributed between nests by workers using the same behaviors as they use to forage. Resources are therefore shared between nests using simple, pre-existing behaviors.

Twitter: @Samellisq

## INTRODUCTION

Resource sharing is a fundamental form of cooperative behavior. The benefits of resource sharing can be direct, such as an increase in the growth or survival of offspring provisioned by a parent (Ydenberg 2007), or more indirect, such as increased access to resources provided by foraging in a group (Waite and Field 2007). In eusocial insect societies, resource sharing behaviors are vital to the survival and fitness of a colony. Only a small proportion of the individuals within a colony are usually involved in the collection of resources, so it is important for the colony to effectively redistribute these resources throughout the rest of the colony, especially to the brood and reproductive individuals. A variety of complex collective behaviors are involved in facilitating, and regulating, within-colony resource redistribution (e.g., Boi et al. 1999; Robinson et al. 2009; Sendova-Franks et al. 2010; Mersch et al. 2013).

For social insects, resource redistribution becomes more complicated if a single colony inhabits several spatially separated nests. This distributed nesting strategy, called polydomy, is common in ants; it is found in at least 150 species and is represented in all the major ant subfamilies (Debout et al. 2007). In a polydomous ant colony, not only do resources have to be redistributed within a single nest but also between nests (Robinson 2014). In social insects, resources are redistributed based on the behaviors of individual workers. Resource redistribution between nests in a polydomous colony must also be based on the distributed behaviors of individual workers.

In polydomous colonies of the red wood ant *Formica lugubris*, resource redistribution between nests occurs along trails of workers traveling between the nests within the colony (Ellis and Robinson 2014). An important resource transported along these internest trails is honeydew (Ellis et al. 2014). Analysis of the structure of the network formed by the nests and internest trails in polydomous wood ant colonies has suggested that they are used to transport honeydew locally, between pairs of nests, rather than through the entire colony (Ellis et al. 2014). This suggests that individual workers are traveling, and transporting resources, along a particular trail between 2 nests, rather than traveling from nest to nest throughout the colony, but it is unknown how this is organized and how this pattern relates to the behavior of individual ants.

Two mechanisms have been suggested for how individual workers could transport resources between nests in polydomous colonies. The first mechanism is based on the idea of a class of internest-transport workers. Transporters are workers specialized at moving resources along a particular internest trail. These workers would transport resources in both directions along the trail, dependent on the abundance of resources in the nests at each end of the trail. These transporters are associated with a particular trail, rather than a particular “home” nest (i.e., the nest a particular worker belongs to and is attempting to benefit). Social insects generally contain many specialized classes of workers (such as foragers and nurses), and so a specialized class of internest transporters might be expected. Indeed workers specialized at transporting resources between polydomous nests have been found in some ant species (*Cataglyphis iberica* Dahbi et al. 1997; *Camponotus gigas* Pfeiffer and Linsenmair 1998) and suggested in red wood ants (Rosengren 1971).

The second possible mechanism is based on internest transport using the same behavior as foraging (McIver 1991). Wood ants foraging for honeydew show a very high degree of route and site fidelity; marked ants have been observed following the same foraging trail to the same foraging site for entire foraging seasons, even after the reward at the end of the trail was no longer present (Rosengren and Sundström 1987; Gordon et al. 1992) or after an artificially extended winter (Rosengren and Fortelius 1986). Indeed, route and site fidelity is a common resource acquisition behavior in a variety of ant species (Tilles and Wood 1986; McIver 1991; Quinet and Pasteels 1996; Gordon 2012). This method of foraging is possible because honeydew is a spatially and temporally stable food source for a red wood ant colony: The aphid colonies providing the honeydew appear to persist within and between years (Rosengren 1977; Ellis S, personal observation). Route and site fidelity could also be used to transport resources between nests in a polydomous colony, with workers based in a particular nest treating other nests in the colony as food sources. In this case, workers would travel from a particular “home” nest to neighboring nests, take the resources they need, and return to their home nest; in the same way, they visit honeydew-producing aphids in the canopy. Under the transporter mechanism, workers on internest trails are balancing resources between 2 nests and are therefore basing their transport behavior on the resources available at 2 nests. In contrast, under the foraging hypothesis, workers are only working to increase the resources in their home nest and are therefore basing their transport behavior on the resources available only at that nest.

**Figure 1:**
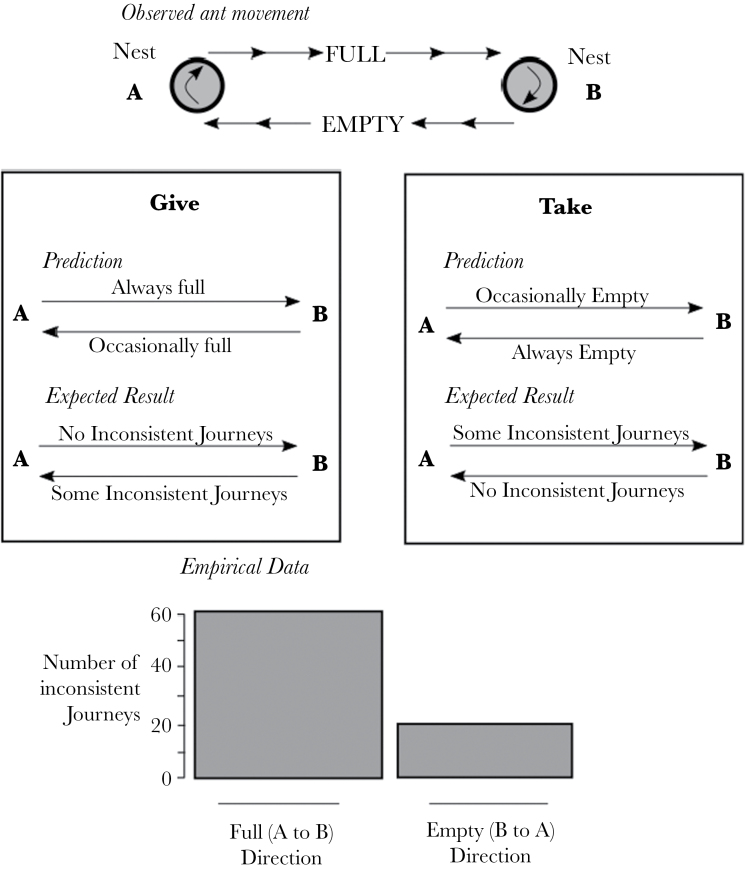
The predications and expected results of the give and take hypotheses, compared with the observed empirical data. Significantly more ants marked as Full (A to B)–Empty (B to A) are inconsistent in the full (A to B) direction than the empty (B to A) directions, as expected under the take hypothesis (AoD^12^, χ^2^ = 19.8, df = 1, *P* < 0.001).

In this study, we marked workers traveling between nests in polydomous *F. lugubris* colonies, to distinguish between these 2 alternative mechanisms. Specifically, we asked 1) Is there a class of internest workers traveling along the trails carrying honeydew in one direction and empty of honeydew in the other direction? 2) Is the direction in which a given worker transports honeydew consistent? 3) Are the honeydew transport behaviors based on ants from a particular nest giving honeydew to neighboring nests or ants from a particular nests taking honeydew from neighboring nests (Table 1; Figure 1)? A specialized transporter class would be expected to show workers redistributing resources in response to local demand or local excesses. If a particular nest has a demand, a transporter may visit nearby nests to take food or conversely, if a particular nest has an excess, workers may take resources from that nest to neighboring nests. This would result in workers transporting resources in both directions along trails in response to local conditions at the nests, or rather, perceived local conditions (individual workers are unlikely to have perfect information). Transport of resources in both directions along a particular trail would manifest itself either as workers constantly carrying resources both ways along a trail or changing the direction in which they transport resources over short timescales. This inconsistency in transport direction is likely to be particularly obvious on trails between nests with relatively even resource levels. In contrast, a worker treating other nests as food sources will consistently transport resources towards her “home” nest regardless of relative local demands.

**Table 1:** Comparison of the expectations and predictions of the give and take mechanisms of resource redistribution between nests

	Give mechanism	Take mechanism
Mechanism	Workers leave their “home” nest **full** travel to a neighboring nest, **give** workers from that nest food, and return to the “home” nest **empty**	Workers leave their home nest **empty**, travel to neighboring nest, take food from workers in that nest, and return **full** to their home nest
Prediction	Few workers will leave the home nest **empty**. However, they may fail to find workers to give their food to and have to return to the home nest **still full**	Few workers will leave the home nest **full**. However, they may fail to find and food to take at the other nest and return to the home nest **still empty**
Expected result	More inconsistent journeys in the direction which the ants were marked as “empty” than the direction which they were marked as “full”	More inconsistent journeys in the direction which the ants were marked as “full” than in the direction which they were marked as “empty”

We predict, therefore, that a transporter class will either travel laden with honeydew in both directions or be inconsistent in the direction in which they do carry resources, along internest trails making it unclear if individual workers are giving food to neighboring nests or taking from them. A foraging worker would, however, be expected to only travel laden in one direction, and for that direction of travel to be consistent, as they take food from neighboring nests.

## METHODS

### Study species and field site

This study was conducted at the Longshaw Estate in the Peak District National Park, UK. There is a large population of *F. lugubris* at the site, with over 900 nests within the 0.95 ha area (Ellis S, personal observation). The habitat at the site contains a mix of deciduous woodland, mixed woodland, open sparsely planted grasslands, and the remains of historic scots pine plantations. There are no other members of the *Formica rufa* group at the site. All mapping, marking, and observations were undertaken when the wood ants were active on internest trails, and, as far as possible, in warm and dry conditions, when ant activity is highest.

### Colonies

**Figure 2:**
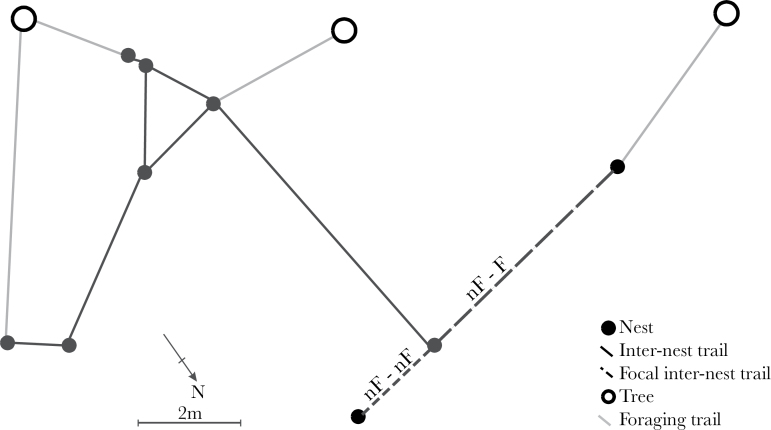
Example of a colony used in this study (colony IV, Table 2). Closed circles are nest, and black lines are internest trails. Open circles are trees, and gray lines are foraging trails. The dashed trails are the experimental trails.

Five polydomous *F. lugubris* colonies with the appropriate trail types for this study (see below) were chosen from the results of a preliminary site survey undertaken in May 2013 (see Table 2 for details). Before the experiment began, we mapped the colonies (by hand), recording the locations of nests; internest trails; foraging trails; and the trees being foraged to (detailed methods, Ellis et al. 2014; e.g., Figure 2). By examining the colony maps, we classified each of the nests within the colonies as “foraging” or “nonforaging,” based on the presence or absence of trails from the nest to a tree (Ellis et al. 2014; Ellis and Robinson 2015). It is important to note that, as the definition of foraging is based only on connections to aphid bearing trees, it does not preclude nonforaging nests from performing other foraging activities such as scavenging and hunting. Internest trails were then classified as between 2 foraging nests (F–F), between 2 nonforaging nests (nF–nF), or between a foraging nest and a nonforaging nest (nF–F). Each experimental colony had 2 trails chosen for trials; one nF–F trail and one either F–F trail (3 colonies) or nF–nF trail (2 colonies). Some colonies had more than 1 trail in a particular category (e.g., several nF–F trails). In these cases, the experimental trail was selected at random from the appropriate trails.

**Table 2:** Details of the 5 colonies used in this study

Colony	Number of nests	Experimental trail types	Relative position of trails	Total number of twice marked ants
I	4	nF–F and F–F	Linked	189 and 118
II	12	nF–F and F–F	Separate	79 and 57
III	11	nF–F and F–F	Separate	104 and 104
IV	9	nF–F and nF–nF	Linked	184 and 147
V	3	nF–F and nF–nF	Linked	94 and 97

“Linked” trails are those where the 2 experimental trails share a nest, whereas “separate” trails are those where the 2 experimental trails are in different parts of the colony and do not share a nest. Number of marked ants refers to the total number ants painted on the nF–F trail and other trail, respectively.

### Assessing load

Honeydew is transported in the crops of ant workers, and in *F. lugubris* storage causes a visible swelling of the gaster (Ellis S, personal observation). Applying gentle pressure to the gaster of a full worker will cause the honeydew stored in the crop to be regurgitated (e.g., Cherix 1987). We use both the visible swelling of the gaster and application of gentle pressure, to assess the presence or absence of honeydew in the gaster of the workers. During preliminary experiments, before the trials, our accuracy at assessing presence or absence of honeydew from visual cues alone was 96% (Ellis S, unpublished data).

### Marking

To investigate how the movement of individual workers between nests facilitates transport of resources through polydomous *F. lugubris* colonies, we individually marked workers as they traveled between nests along the chosen trails. Workers were marked with “Uni-Paint” marker pens (Mitsubishi Pencil Co. UK Ltd). Paint marking has been used extensively in previous studies of ants with no disruption to their behavior (Beverly et al. 2009; Franklin et al. 2010; Chen and Robinson 2013); we observed no overt changes in behavior or increased mortality in the marked ants. We used a unique pattern of colors for each experimental trail within the colony.

On the first day of a trial, a direction along the trail was randomly chosen. Five hundred workers traveling in the selected direction were painted as they passed along the trail. Ants were chosen for marking by selecting the first ant to pass a defined point in the direction of interest, and continue traveling in that direction for 5cm past that point. Once a given ant was marked, it was replaced onto the trail, and the next ant to pass the defined point was then selected for marking. By marking ants as they passed a particular point, we aimed to select a representative sample of the ants traveling along the trail, without biasing our marking toward different sized ants or workers with different rates of travel. The painting was done in batches of 100 ants over the course of 1 or 2 days to minimize time of day effects. Workers were painted one of 2 colors on the thorax; one color if an ant was laden with honeydew (full) and another if the ant carried no honeydew (empty). Load was assessed by applying pressure to the gaster of the ant to observe for regurgitation of honeydew: this ensured a high level of accuracy for the marking. The following day, all painted ants passing in the opposite direction (to that painted the day before) were assessed for load, and then painted on the head as either full or empty. This second painting session was conducted in seven 30-min intervals over the course of 1 day, to control for time of day effects (painting totals: Table 2). The result after both marking sessions was that each trail had a cohort of workers marked as full or empty on both the thorax (representing load in one direction) and the head (representing load in the other direction).

### Observation

For 5 days immediately following the completion of painting, the trails were observed for 30min per day. During the observations, for all marked ants passing a particular point, we recorded the direction of travel, painted pattern, and presence or absence of honeydew load. Load was assessed by visual examination of the workers, rather than by applying pressure, to minimize further disruption to the workers. These sessions of trail observation are hereafter referred to as the “observation period.”

### Straying

Previous work has suggested that ant workers in polydomous *F. lugubris* colonies are only traveling locally, between pairs of nests, rather than moving freely through the whole colony (Ellis et al. 2014). To confirm that workers are only moving locally, we estimated the rate at which ants stray from a given internest trail onto other trails in the same colony. Straying frequency was estimated by randomly selecting a trail sharing a nest with the experimental trail each day and observing that trail for 10min, noting the direction of travel, paint pattern, and honeydew load of any doubly painted ants on that trail. The type of trail workers stray onto may help reveal the role they are playing in the resource redistribution mechanism. Workers traveling through the colony to find food are expected to stray preferentially onto foraging trails, as the trees are the food sources for the colony.

### Statistical analysis

Analysis was performed using generalized linear mixed effect models (GLMMs) in the “lme4” package in R (R Development Core Team 2011). The response variables and fixed effects changed based on the question being investigated. Unless noted otherwise, only a single fixed effect was used. Additionally, colony of origin and day of the experiment were used as nested random effects to control for repeated observations of the same trail. Further details of the statistical models used are found in the Supplementary Material: The superscript in the text refers to the row of the table that contains the details (Supplementary Material).

All GLMMs used a binomial error structure with a logit link function. Tests of significance were performed by a chi-square analysis of deviance (AoD); the results of these tests are reported in the text. The AoD compares the full GLMM to the same GLMM but with the variable of interest removed. A significant difference between these 2 models indicates a significant effect of the variable in explaining the data. Where the significance of a particular variable is reported, the values are taken directly from the model.

## RESULTS

Our results revealed that a significant majority of workers on internest trails travel in one direction full and the other empty and that these workers consistently carry resources in this single direction over the course of the 5 days of the experiment. In addition, the results suggest that the internest resource exchange mechanism is based on workers taking resources from, rather than giving resources to, neighboring nests.

### Directionality

Our results show that there is a class of workers traveling full in one direction and empty in the other (Table 3). Overall, 742 of 1173 (63%) of marked workers had different loads in each direction, whereas only 80 (7%) were full in both directions and 351 (30%) were empty in both directions. The workers traveling full in one direction and empty in the other can be considered to be directional workers, carrying honeydew in one direction but not the other. There is a significant negative association between load in each direction (AoD^1^, χ^2^ = 48.1, degrees of freedom [df] = 1, *P* < 0.001), meaning that there are significantly more directional ants than ants traveling with the same load in both directions. The different colonies used in the experiment do have significantly different proportions of directional ants (AoD^2^, χ^2^ = 34.2, df = 4, *P* < 0.001; Supplementary Material 2). This is driven by a significantly higher proportion of directional ants in colony I (GLMM^2^, *z* = −2.981, *n* = 1173, *P* < 0.01). Proportion of directional workers also varies significantly depending on trail type (AoD^3^, χ^2^ = 24.3, *n* = 1173, df = 2, *P* < 0.001). There are a significantly lower proportion of directional workers on nF–nF trails than on other trail types (GLMM^3^, *z* = 2.994, *n* = 1173, *P* < 0.01). This is likely to be particularly influenced by the observation that one of the 2 tested nF–nF trails (on colony V) has a very low proportion of directional workers. The other tested nF–nF trail (on colony IV) shows a similar proportion of directional workers to other trails.

**Table 3:** Total number of ants painted in each category during the course of 10 trials

		AB direction	
		Full	Empty
BA direction	Full	80	516
	Empty	226	351

The trails are between nests arbitrarily named “A” and “B.” The AB direction indicates ants traveling from nest “A” toward nest “B,” and BA direction is ants traveling in the opposite direction (from nest “B” to nest “A”).

### Consistency

All journeys in the observation period were classified as either “consistent” or “inconsistent.” A journey is consistent if the honeydew load of an observed ant matches that which it was initially marked transporting. For example, an ant initially marked as full when traveling in a particular direction is acting consistently if, when later observed traveling in that same direction (during the observation period), it is transporting honeydew; conversely, it is acting inconsistently if it is empty of honeydew.

**Figure 3:**
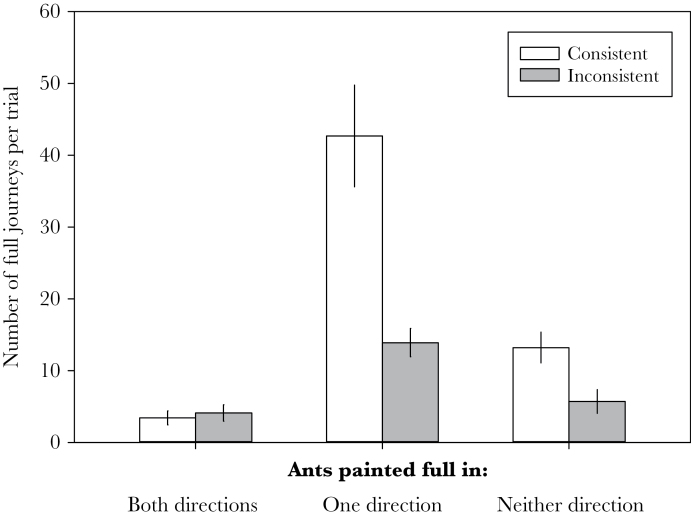
Mean (± standard error) number of ants behaving consistently and inconsistently over the course of 5 days of observation based on their originally painted pattern. Workers are considered consistent if their observed behavior matched their painted behavior. Ants originally painted as laden with honey dew (“full”) in one direction (and empty in the other) were significantly more consistent in their behavior than either the ants traveling painted as laden in both directions or those painted as empty in both directions.

Over the course of all 10 trials, 693 of 927 (75%) of observed journeys were consistent; this is significantly higher than the number of inconsistent journeys (AoD^4^: χ^2^ = 11.7, df = 1, *P* < 0.001). Consistency of behavior varies significantly based on painted pattern of the ant (AoD^5^, χ^2^ = 34.1, df = 2, *P* < 0.001; Figure 3); ants painted as traveling full in one direction only are significantly more consistent than both those painted as full in both directions (GLMM^4^, *z* = −5.9, *n* = 927, *P* < 0.001) and those painted as empty in both directions (GLMM^4^, *z* = −2.1, *n* = 927, *P* = 0.04).

This consistency does not vary either with trail type (AoD^6^, χ^2^ = 3.5, df = 2, *P* = 0.17) or over the course of the 5 days of the experiments (AoD^7^, χ^2^ = 4.8, df = 4, *P* = 0.31). Interestingly, however, if ant painted type is introduced as an interacting fixed effect then there is a significant effect of day on the proportion of consistent ants (AoD^8^, χ^2^ = 43.0, df = 5, *P* < 0.001). The interaction between day and ant painted pattern is significant (AoD^9^, χ^2^ = 4.99, df = 2, *P* = 0.03); this is driven by a significant negative interaction between ants that were marked as traveling empty in both directions and day of the experiment (GLMM^9^, *z* = −0.34, *n* = 927, *P* < 0.001). This suggests that, over the course of the experiment, ants initially traveling empty in both directions became significantly less consistent in their behavior.

### Straying

Ants strayed from their painted trail at an average rate of 1.16±0.27 (mean ± standard error) per 10-min observation (8.1% ± 13.4 of the number of marked ants observed on the focal trails). Ants with differing marked patterns (e.g., full in both directions, full in one direction, or full in neither direction) were equally likely to stray from the focal trail (AoD^10^, χ^2^ = 1.7, df = 2, *P* = 0.44). Ants were equally likely to stray onto other internest trails as onto foraging trails (AoD^11^, χ^2^ = 0.78, df = 1, *P* = 0.38).

### Give or take?

The results above suggest that there is a class of workers consistently traveling one direction along an internest trail full and in the other direction empty. However, what is not clear from these results is whether resource exchange is based on workers from one nest carrying food to their neighbors (a give mechanism) or workers from a particular nest taking food from their neighbors (a take mechanism).

Given that workers are acting consistently (see above), some predictions of the behavior can be made (Table 1; Figure 1). The give and take mechanisms lead to differing expected patterns of inconsistency along a trail depending on the mechanism in place (Table 1; Figure 1). By comparing ants painted pattern to the proportion of inconsistent journeys in either direction, it is possible to differentiate between the 2 mechanisms. We found that that 75.6% (99/131) of inconsistent journeys made by ants marked carrying resources in only one direction (i.e., full-empty or empty-full ants) were inconsistent in the direction in which they were originally marked as laden with honeydew (the “full” direction). The proportion of inconsistent journeys is significantly higher in this “full” direction than in the direction they were marked as empty (full-empty marked ants; AoD^12^, χ^2^ = 19.8, df = 1, *P* < 0.001: empty-full marked ants; AoD^13^, χ^2^ = 14.0, df = 1, *P* < 0.001). A greater proportion of inconsistent journeys in the full direction is expected under the “take” mechanism (Table 1; Figure 1).

### Resource movement

**Figure 4:**
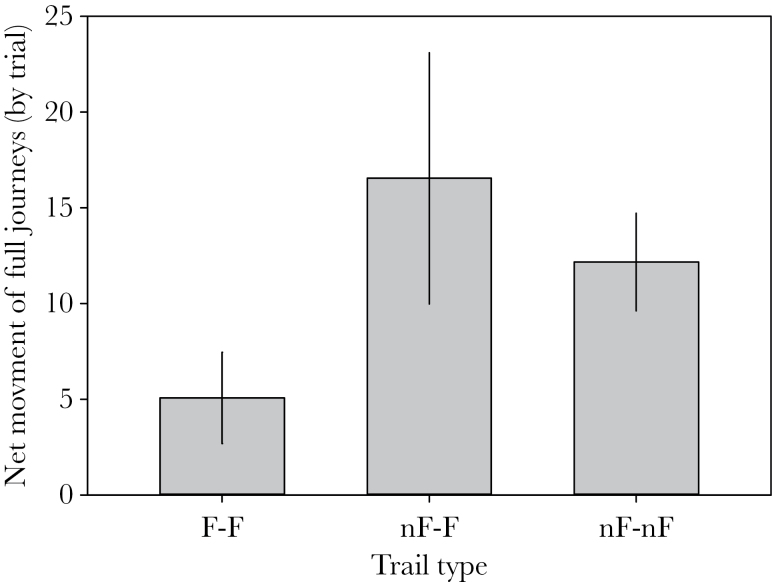
Mean (± standard error) number of honeydew-carrying (“full”) journeys along internest trails of different types. The net movement of resources is significantly lower on trails between 2 foraging trails (F–F) than on either trails from a nonforaging nest to a foraging nest (nF–F) or trails between 2 nonforaging nests (nF–nF), suggesting a less uneven exchange of resources on F–F trails.

With one exception, resource movement is toward the nonforaging nest along nF–F trails. However, it is interesting to note that, even though net-resource movement was toward the nonforaging nests, 31.9% (180/564) observed journeys on the nF–F trails were still transporting resources from the nonforaging toward the foraging nest. The net movement of resources along trails is significantly lower on F–F trails than on other trail types (AoD^14^, χ^2^ = 18.5, df = 2, *P* < 0.001; Figure 4), suggesting that resource flow is significantly less uneven on F–F trails than other trail types. On both the nF–nF trails, net-resource flow is from a nest that has another trail to a foraging nest toward a nonforaging nest that had no trails to foraging nests. The nF–F trail exception is colony II, and in this colony, when re-examined later in the season, the nonforaging nest had begun foraging, which may explain its unusual behavior.

## DISCUSSION

Our study shows that mechanism of honeydew exchange between nests of polydomous *F. lugubris* colonies is based on individual workers treating other nests of the colony as food sources. This is supported by the results showing 1) a cohort of workers travel along internest trails in one direction full and the other empty, 2) they do this consistently and do not change role or direction of transport over a short timescale, and 3) workers are taking food from neighboring nests, rather than giving food to their neighbors.

In general, when foraging, ant workers leave the nest, collect food, and return to the nest transporting this resource. This process necessarily results in the majority of foragers traveling in one direction without carrying food and the other direction carrying food. If workers are treating other nests in a polydomous colony as food sources, a similar pattern of transport would be expected. This is the pattern we found in the internest workers of the *F. lugubris* colonies, with significantly more workers observed traveling in one direction full and the other empty than traveling with the same load in both directions. In this study, we have investigated only the movement of honeydew between nests, but a system based on foraging to other nests would also work as a method to redistribute other resources. For example, as the majority of protein for red wood ant is provided by hunting and scavenging in the canopy (Cherix 1980; Cherix 1987; Rosengren and Sundström 1991; Robinson et al. 2008), this is likely to lead to nests with honeydew foraging trails also acting as stable sources of protein, and therefore amenable to an internest-transport system based on foraging. Seven percent of workers were marked as traveling full in both directions. The role these workers are playing is unclear; they could simply be lost or have mistakenly left the nest while still full. If these 7% of ants are lost or mistaken ants, a similar proportion of workers leaving along the nest to forage would be expected to be full. Further work is necessary to establish if this is the case.

Foraging wood ants show high levels of site allegiance and route fidelity; they consistently travel from the same nest along the same foraging route, in many cases independently of the presence or absence of a resource at the end of the route (Rosengren 1971; Gordon et al. 1992). If a similar pattern is used to transport resources between polydomous *F. lugubris* nests, it is to be expected that the workers marked as transporting resources in one direction will continue to transport resources only in that direction, and not change their role, or direction of transport, over time. In contrast, if resource transfer is undertaken by a specialist internest-transporter class, the direction of resource transfer is expected to vary, especially between nests where resource flow is relatively even. For example, on a trail between 2 foraging nests a transporter might reach one of the nests, find an excess and therefore transport honeydew to the other nest. At the second nest they might, again, find an excess and therefore transport honeydew back to the original nest resulting in a relatively even resource flow and a cohort of workers transporting in both directions along a trail. The reverse is also true, if transporters find a need at a particular nest they will react by going to the neighboring nest and collecting food to return to the original nest. In this study, we found that workers, especially directional workers, show a high degree of consistency in their behavior, indicating that they consistently transporting resources in a single direction. This applies even on trails where the net resource flow is relatively even (F–F trails), strongly suggesting that internest resource transfer is based on high route fidelity and site allegiance with workers treating other nests of the colony as food sources. On trails between nests with relatively even resource availability an individual transporter, in a transporter-class system, would be expected to show very inconsistent directions of resource transfer, responding to small, or temporary, perceived differences in supply or demand.

Our results agree with previous working suggesting that workers on internest trails in *F. lugubris* colonies travel locally, between pairs of nests, rather than traveling freely through the whole colony (Ellis et al. 2014). The low frequency of ants straying between trails suggests that workers are only traveling along a single internest trail, not through the whole colony. As the trees are the food sources, we expect that if workers are traveling freely through the network to find resources they would preferentially travel to foraging trails. The result that workers are equally likely to stray onto internest trails as onto foraging trails, suggests that workers are not preferentially traveling to foraging trails. This in turn suggests that the strays may simply be lost or mislabeled ants, rather than part of the resource redistribution system.

Details of the development of this resource redistribution mechanism are suggested by the observation that workers marked traveling empty in both directions along trails become less consistent in their behavior over the course of the experiment, meaning that they have begun transporting resources. It could be that these workers are inexperienced foragers who are in the process of being recruited to a source of honeydew. Over the course of the observation period some of these inexperienced workers may have become recruited to the trail under observation and therefore become directional transporters. In our study, this would manifest as the workers marked traveling empty in both directions beginning to transport resources and therefore becoming inconsistent with their painted pattern, which is what we have found. Inexperienced foragers being recruited to foraging trails have been observed in wood ants (Gordon et al. 1992) but not investigated in the context of internest trails. The recruitment of inexperienced or naive workers to a particular task is widely reported in social insects generally. In many cases, the recruited individuals revert to being naive again either when the task has been completed (e.g., Langridge et al. 2004) or after a variable amount of time (e.g., Seeley and Buhrman 1999), whereas, in the case of foraging wood ants, this recruitment appears to be permanent, at least for a subset of the foragers (Gordon et al. 1992; Lamb and Ollason 1994; Ellis S, unpublished data). Our results suggest that recruitment of inexperienced foragers to particular trails (in this case internest trails) may also apply to internest *F. lugubris* workers, but further investigation is needed to fully understand the origins of the recruited ants and the method of recruitment.

Our study has shown that this consistent behavior is directed toward taking food from neighboring nests. This is an interesting result because it suggests that nests are acting almost independently to collect honeydew. Although allowing workers from other nests to take food is a form of passive support, nests appear to offer very little active support to each other. This is highlighted by the observation that even on trails between nF–F nests there is some honeydew carried from the nonforaging nest toward the foraging nests. Polydomous, polygynous, wood ant colonies usually expand by budding: Workers and queens leave the nest on foot and found a new nest nearby (Ellis and Robinson 2014). Shared descent is likely to lead to strong genetic spatial clustering (Sundström 1993; Chapuisat and Keller 1999; Gyllenstrand and Seppä 2003; Pamilo et al. 2005; Sundström et al. 2005). Resource sharing between nests is, therefore, not only likely to directly benefit individuals in the nest taking resources, but is also likely to indirectly benefit individuals in the nest allowing their resources to be taken.

Division of labor is a defining feature of eusociality (Hölldobler and Wilson 1990): At the simplest level, this involves transferring resources from a sterile worker caste to a reproductive caste. Within this basic eusocial framework, there are many examples of more complex division of labor, between foragers and nest workers, for example, or between different classes of foragers. Active transfer of resources between these classes and castes is regularly observed and is necessary for the proper functioning of the colony. It might be expected that a similar mechanism is present in polydomous colonies, perhaps with larger foraging nests supporting other smaller nonforaging nests in the colony by actively supplying other nests of the colony with excess honeydew. The fact that this is not the case indicates that nests of the colony act almost independently when collecting honeydew, treating the rest of the colony as food sources. The only concession nests in a polydomous colony make to the rest of the colony is to allow their honeydew to be taken. This does not preclude different nests in the network performing different roles (some forage and others do not, e.g.; Ellis and Robinson 2015), indeed, *F. aquilonia* workers have been found to differ in size in different nests from the same colony perhaps suggesting a differing role (Kennedy et al. 2014). However, our results suggest that this division of labor is not due to a colony-level strategy (with some nests as, e.g., foraging specialists), rather it is based only on the properties and environment of individual nests.

The advantage of this “foraging” mechanism is likely to be its simplicity. It requires no colony-level organization, and results from simple self-organization in worker behavior. Newly foraging ants begin as inexperienced foragers and are recruited to a food source with an excess, either a foraging tree or another nest, and then transfer resources consistently from that food source back to their home nest. A similar system has been found in some monodomous species, where colonies treat the nests of other colonies as food sources; stealing resources in a form of intraspecific kleptoparasitism (Breed et al. 1990; Yamaguchi 1995). The main difference between the intraspecific kleptoparasitism system and the polydomous nest system is that, whereas in the monodomous kleptoparasitic colonies this causes an aggressive response from the workers in the nest being taken from, within a polydomous colony there is no aggressive response to the intruders. A mechanism of resource redistribution within polydomous colonies based on workers treating other nests in the colony as food sources has been previously suggested, and modeled, in polydomous ants (McIver 1991; Schmolke 2009; Cook et al. 2013), but to our knowledge, this is the first example of such a mechanism being observed in natural populations. Agent-based models of polydomous ant colonies have shown that workers treating other nests of the colony as food sources can result in resource redistribution through the whole colony (Schmolke 2009; Cook et al. 2013).

The mechanisms of resource acquisition and redistribution for a species are likely to be closely linked to the type of resource which they are attempting to exploit (Lanan 2014). For species relying on a temporally and spatially stable food source, like honeydew is for *F. lugubris*, the mechanism of resource acquisition may not need to be flexible to short-term changes in resources availability (if there are any). Instead, it is likely to be more important for the ants to adjust to longer-term trends in resource availability (Gordon et al. 1992), which a mechanism based on internest foragers would be capable of doing. In contrast, a mechanism based on either a specialist transporter class or of nest giving food to its neighbors, requires the ability of workers to 1) recognize the difference between food sources and other nests and 2) assess the relative need of other nests in the network for honeydew and transport resources according to this need. Such a system may be able to adjust more accurately to short-term changes in demand, but is much more computationally complex. This highlights the importance of timescale when investigating a species’ foraging ecology. For species with low resource reserves that are dependent on unpredictable food sources, all nutrient sources, when they appear, are likely to be critical to survival (e.g., Rogers and Smith 1993; Bonter et al. 2013). In contrast, for species foraging on large and predictable food sources, short-term changes are probably less important than the long-term trends which, in the case of many social insects, can be adjusted to over the scale of generations of workers.

A simple resource redistribution mechanism, such as that described here, may help explain the plasticity shown by *F. lugubris* and other *F. rufa* group species in their nesting strategy. If nests forage to neighboring nests in the same way they forage to food sources, then the workers are using pre-existing resource acquisition behaviors. This means that there is less behavioral innovation required for a colony to move from being monodomous to polydomous. Rather than a complete change in life history, involving new behaviors, polydomy can in fact be viewed as a continuation of existing behaviors requiring few innovations. Polygynous wood ant colonies usually reproduce by budding (Rosengren and Pamilo 1983; Ellis and Robinson 2014). If when a new daughter nest is budded from an existing “mother” nest, that daughter nest uses the mother nest as a food source and the mother uses the daughter as food source, a polydomous system has developed out of pre-existing foraging behaviors.

Beyond the *F. rufa* group, in ants generally, polydomy has probably evolved many times independently (Debout et al. 2007). A mechanism of resource exchange based on pre-existing behaviors may help facilitate this repeated evolution of the same nesting strategy. Polydomy may, therefore, provide an interesting variation on the idea of behavioral convergence. Behavioral convergence is the repeated evolution of the same behavioral traits by species in similar environments (e.g., Blackledge and Gillespie 2004; Stoks et al. 2005; Johnson et al. 2010; Alejandrino et al. 2011). In the case of polydomy, rather than the behavior evolving multiple times in response to similar ecological conditions, the same behavior may have evolved multiple times in response to a variety of different ecological conditions, due to an inherent predisposition for the behavior. The diversity of habitats, life-history strategies, and diets associated with polydomy may be a result of this inherent behavioral predisposition. Repeated evolution of polydomy may also be a factor in the diversity of internest transportation strategies that have been observed in polydomous colonies. A mechanism of internest resource redistribution based on foraging to other nests could evolve into a transporter class, given the right life history and ecological circumstances. For example, transporters have been found in polydomous *C. gigas* colonies (Pfeiffer and Linsenmair 1998). *Camponotus gigas* feeds primarily on honeydew (Pfeiffer and Linsenmair 2000), much like red wood ants. However, unlike polydomous wood ants, *C. gigas* is monogynous (Pfeiffer and Linsenmair 1998), which is likely to profoundly affect its life history, and may make a transporter-class beneficial.

This study has found that the resource redistribution through a spatially separated wood ant colony is achieved by the same behaviors used for foraging. Our results highlight how resources can be moved through a complex system based on simple, self-organized behaviors.

## SUPPLEMENTARY MATERIAL

Supplementary material can be found at http://www.beheco.oxfordjournals.org/

## FUNDING

S.E. acknowledges funding from NERC and the National Trust. E.J.H.R. acknowledges funding from the Royal Society.

## Supplementary Material

Supplementary Data
