# Supplementary material for: Internest food sharing within wood ant colonies: resource redistribution behavior in a complex system
Source: Behav Ecol. 2015 Nov 30;27(2):660–8. doi: 10.1093/beheco/arv205 (PMC4797383; doi:10.1093/beheco/arv205)
Supplement: Supplementary Data [file supp_27_2_660__index.html]

Internest food sharing within wood ant colonies: resource redistribution behavior in a complex system — Internest food sharing within wood ant colonies: resource redistribution behavior in a complex system — Supplementary Data 

# Internest food sharing within wood ant colonies: resource redistribution behavior in a complex system

## Supplementary Data

Data files

- Supplementary Data - Supplementary Data
- Supplementary Data - Supplementary Data
