# Supplementary material for: Internest food sharing within wood ant colonies: resource redistribution behavior in a complex system
Source: Behav Ecol. 2015 Nov 30;27(2):660–8. doi: 10.1093/beheco/arv205 (PMC4797383; doi:10.1093/beheco/arv205)
Supplement: Supplementary Data [file supp_arv205_supplem_2.docx]

**Supplementary Material 2**

Number of directional (different load in each direction) and non-directional (same load in each direction) ants marked by colony. Values show the mean ± SE of the two trials undertaken on each colony. There are significantly more directional journeys than non-directional ants (AoD^1^, χ^2^=48.1, *df*=1, *p*<0.001). There is a significant difference between proportion of directional ants in each colony (AoD^2^, χ^2^=34.2, *df*=4, *p*<0.001), driven by a significantly higher proportion of directional journeys in colony I (GLMM^2^, *z*=-2.981, *n*=1173, *p*<0.01).
