# Supplementary material for: Internest food sharing within wood ant colonies: resource redistribution behavior in a complex system
Source: Behav Ecol. 2015 Nov 30;27(2):660–8. doi: 10.1093/beheco/arv205 (PMC4797383; doi:10.1093/beheco/arv205)
Supplement: Supplementary Data [file supp_arv205_Supplementary_Material.docx]

**Supplementary Material**

Details of the statistics used in the study. # refers to the superscript number in the text. The Dependent variable, fixed effects and random effects describe the GLMM used, all used a binomial error structure. In all tests errors were heteroscedastic and were not overdispersed. χ^2^, *df* and *P* describe the results of an analysis of deviance, which compares the model to a null model which lacks the variable of interest.

| **#** | **Response Variable** | **Fixed Effect(s)** | **Random Effect(s)** | **χ^2^** | ***df*** | ***P*** |
| --- | --- | --- | --- | --- | --- | --- |
| 1 | Direction 1 load | Direction 2 load | colony/trail type | 48.1 | 1 | <0.001 |
| 2 | Direction 1 load | Direction 2 load , colony | trail type | 34.2 | 4 | <0.001 |
| 3 | Direction 1 load | Direction 2 load , trail type | colony | 24 | 2 | <0.001 |
| 4 | Consistent journeys | Inconsistent journeys | colony/day, trail type | 11.7 | 2 | <0.001 |
| 5 | Consistent vs. Inconsistent journeys | Paint pattern | colony/trail type/ day | 34 | 3 | <0.001 |
| 6 | Consistent vs. Inconsistent journeys | Paint pattern, trail type | colony/day | 35.5 | 2 | <0.001 |
| 7 | Consistent vs. Inconsistent journeys | Day | Ant type\| colony/trail type | 4.8 | 4 | 0.31 |
| 8 | Consistent vs. Inconsistent journeys | Day x Ant type | Colony/ trail type | 43.0 | 5 | <0.001 |
| 9 | Consistent vs. Inconsistent journeys | Day x Ant type | Colony/ trail type | 5.0 | 2 | 0.03 |
| 10 | Stray journeys | Ant type | Colony/ trail type/ day | 1.7 | 2 | 0.44 |
| 11 | Stray journeys | Trail type | Colony/ trail type | 1.8 | 1 | 0.18 |
| 12 | Consistent vs. Inconsistent journeys | Direction | Colony/ trail type/ day | 19.8 | 1 | <0.001 |
| 13 | Consistent vs. Inconsistent journeys | Direction | Colony/ trail type/ day | 14.0 | 1 | <0.001 |
| 14 | Flow AB direction vs BA direction | Trail type | Colony/day | 18.5 | 2 | <0.001 |
